# The Southern Surgical and Gynecological Association

**Published:** 1889-12

**Authors:** 


### THE SOUTHERN SURGICAL AND GYNECOLOGICAL ASSOCIATION.

SECOND ANNUAL MEETING HELD IN NASHVILLE, TENNESSEE. NOVEMBER 12, 13 AND 14, 1889.

### FIRST DAY—MORNING SESSION.

November 12th.

The Association convened in the Senate Chamber and was called to order at 10 A. M. by the President, Hunter McGuire, of Richmond, Va.

Prayer was offered by Rev. R. L. Cave.

The report of the Committee of Arrangements was made through its Chairman, J. R. Buist, M. D., of Nashville.

Dr. R. B. Maury, of Memphis, Tenn., contributed a paper entitled

### REPORT OF GYNECOLOGICAL WORK WITH ESPECIAL REFERENCE TO METHODS.

The paper presents a brief summary of the more important portion of his operative work during the past year. With four exceptions all the operations reported were done in a private hospital, built especially for the purpose and under his own control. The summary embraces twenty-one laparotomies for the removal of ovarian tumors, or of the uterine appendages, or for the relief of obscure diseases within the abdomen; two laparotomies for ectoptic gestation; twenty-eight operations for laceration of the cervix; fourteen perineal and vaginal prolapse operations; five rectoplasties; four anterior colporrhaphies.

Paper discussed by Drs. Wathen, Price, Stone, Potter and Johnston.

Dr. W. O. Roberts, of Louisville, Kentucky, read a paper on

### DIRECT HERNIOTOMY, in which he reported ten cases and did eleven operations. Seven cases occurred in females, three in males. Three were cases of umbilical hernia, three were femoral, three inguinal, and one ventral. Six of the operations were done during strangulation, while five were performed for troublesome irreducible hernias. In the six cases of strangulated hernia, the sexes were equally represented. Three were inguinal, two femoral and one umbilical. In five of them the operation for radical cure was done. The remaining case was one of strangulated hernia, where stercoraceous vomiting existed for eight hours before it was seen. General peritonitis was evident at the time of the operation. Considerable reddish material escaped when the contents of the sac were returned. Death followed in thirty-six hours.

The four cases of irreducible hernia were all in females. Two were umbilical, one femoral and one ventral. In one of the former a second operation was made necessary by the hernia recurring at the end of six months. The tumor also reformed at the end of ninety days, in the case of femoral hernia. The remaining cases continue well to the present time.

In all the cases the sac was first cleanly dissected out and not opened until all hemorrhage was entirely checked. Both in the ventral and umbilical hernias the integument and fascia were divided by an elliptical incision. After opening the sac all adherent omentum was tied with cat-gut and excised. The remaining contents were then returned into the cavity. In one case of umbilical hernia the neck of the sac was tied close to the margins of the opening, and cut away immediately in front of the ligature. The stump was then fastened tightly in the opening with silkworm gut sutures, and the wound finally closed by interrupted sutures of the same material. In this case no suppuration occurred, but the hernia recurred at the end of six months. In both the other umbilical and in the ventral hernias, the neck of the sac was excised on a level with the abdominal opening and sutured with cat-gut. The opening itself, after its edges were freshened, was closed with the continued suture of chromatized cat-gut in the ventral hernia, and aseptic corded silk in the umbilical cases. The superficies were brought together by silk sutures, which were continued down to the aponeurosis.

In the femoral hernias he adopted the practice of Mitchell Banks. In the cases of direct inguinal hernia, after tying the neck of the sac just within the ring, and removing all in front of the ligature, the ring was closed with cat-gut sutures.

In the cases of oblique inguinal hernia, he did Mr. Ball's operation, which consists in freeing the neck of the sac up to the internal ligature, then twisting the neck upon itself to effect closure of the peritoneal orifice, and finally stitching the stump to the pillars of the ring to guard the neck against untwisting itself.

Dr. Virgil O. Hardon, of Atlanta, Georgia, read a paper on

THE ABORTIVE TREATMENT OF ACUTE PELVIC INFLAMMATION, which appears in this number of the JOURNAL.

Dr. Joseph Price, of Philadelphia, said that his experience was a little different from that of the essayist. Relief from salines and the aspirator, in the cases reported, may have been justifiable, but pus tubes, in every instance, call for abdominal section. Dr. Price then exhibited some pus tubes in connection with his remarks, one of which was seven inches in length, and called attention to their frequency in cases of pelvic inflammation.

Dr. George J. Engelmann, of St. Louis, Mo., said that there was a fascination in the results achieved by Dr. Price and other operators, but thought the pendulum was swinging too far in that direction. He could indorse the surgical treatment advocated by Dr. Price in dealing with cases of pelvic inflammation which result from salpingitis, or where there was pus in the tubes. Many of the cases which were formerly called pelvic cellulitis are the sequences of salpingitis or pus in the tubes, and he was satisfied that there are cases which exist without salpingitis.

Dr. W. G. Ewing, of Nashville, said that when he began to practice medicine he was on the conservative side, but is now satisfied that many of the cases which come under his observation could have been dealt with surgically and successfully. He favored early operative interference, and said the surgeon should not delay in such cases, for by so doing additional adhesions were apt to form, with extension of the inflammatory process.

Dr. Hardon, in closing the discussion, said he feared he had been misunderstood, but would say that pus tubes admitted of but one rational treatment, and that was their removal. He invariably recommends that this be done, but what should the surgeon do with those patients who will not permit an operation? As regards pelvic cellulitis without disease of the tubes, he could corroborate the statement of Dr. Engelmann, and called attention to two cases which he reported last year to the Association which verified this fact. In these cases he aspirated and withdrew several drachms of serum from the cellular tissue. Subsequent examination revealed absolutely no disease of the tubes. He has examined these patients from time to time, but not the slightest evidence of tubal disease could be found, and there has been no recurrence of the pelvic inflammation.

Dr. W. D. Haggard, of Nashville, read a paper on

THE IMPROVED CÆSAREAN SECTION VERSUS CRANIOTOMY, which will appear in the JOURNAL.

### FIRST DAY—AFTERNOON SESSION.

Dr. W. W. Wathen, of Louisville, Kentucky, read a paper on

THE TREATMENT OF ECTOPIC PREGNANCY.

He said in order to adopt the best treatment in ectopic pregnancy, it was necessary to know its correct pathology. Failure in this particular has resulted in a variety of methods of treatment, and gynecologists of good ability differ widely in their views. But physicians who recognize that ectopic pregnancy is probably always tubal in its origin are united in their opposition to the use of electricity or other means to destroy fœtal life. They prefer the removal of the gestation sac by laparotomy. He referred to the fact that Thomas adheres pretty much to the old classification of Parry and DeZeimeris, and that he speaks of an "impregnated ovum attaching itself primarily to the peritoneum, and of a fœtus and placenta entering the peritoneal cavity by rupture and developing there." He denied the possibility of an ovum attaching itself primarily to the peritoneum, and said that the placenta could not become separated from the tube and *then* become attached to other structures. The ovum must be held immovably in one position before its villi can penetrate the tissues. The placenta may become slowly separated from its tubal attachments and fasten itself to other structures by making epiphytic inroads upon the abdominal walls or viscera by stripping off the peritoneum.

He did not believe that a classification, based upon old statistics of *post mortem* examinations, could be correct, because these examinations were made by men not trained in pathological and microscopical research, who could not accurately distinguish the tissues often matted together and entirely changed in physical appearance.

He divided the treatment of ectopic pregnancy as follows :

1. The treatment before primary rupture of the tube.

2. The treatment after rupture of the tube into folds of the broad ligament, and before the period of fœtal viability.

3. The treatment where the sac ruptures into the peritoneal cavity.

4. The treatment after fœtal viability, and at the full period of gestation.

5. The treatment after death of the fœtus, at or before the full period of gestation.

He did not believe that a correct diagnosis is ever probable before primary rupture, and in no instance would he use electricity to destroy fœtal life. He performed laparotomy, and claimed that the immediate and subsequent results would be better. He said that while Dr. Harbert in 1849, Kiwisch in 1857, and Dr. Rodgers in 1867, had suggested the removal of the sac in tubal rupture, he was probably the first to recommend its removal, if the diagnosis is made before rupture. If the tube has ruptured into the folds of the broad ligament and pregnancy has continued four and a half or five months, he advised expectancy, and to operate after fœtal viability, but before the beginning of false labor. He advocated the removal of the placenta aud fœtal membrane, where it is possible to do so, and control hemorrhage by ligation *en masse* at the proximate end of the large vessels. If this cannot be done, it is best to leave the placenta to be absorbed and close the abdominal wound aseptically after the fashion recently described by Mr. Tait.

This paper was followed by impromptu remarks on Perineorraphy, by Dr. A. W. Johnson, Danville, Kentucky.

Dr. Jos. Price, of Philadelphia, contributed a paper on

### PUS IN THE PELVIS AND HOW TO DEAL WITH IT.

By pus in the pelvis he meant pus that has its *fons et origo* in the pelvic organs or their investment. The rarer causes of pus in the pelvis may be said to be (1) carious bones, psoas abscess; (2) traumatism, sloughing, results of electricity, direct violence, etc.; (3) foreign bodies, as extra-uterine bones, etc.

The general rule is that pus in the pelvis is always the result of diseased conditions of the uterine appendages, whether it occurs as a result of a ruptured extra-uterine pregnancy, a suppurating ovarian or dermoid cyst, or salpingitis caused by gonorrhœa, parturition, dirty instruments, electricity or what not. In general, then, when the surgeon finds pus in the pelvis he will find its origin in the uterine appendages. He had seen pus discharging from the rectum, from the bladder, the umbilicus, and from the vagina. He has seen psoas abscess, perforating appendicitis, idiopathic peritonitis and typhoid fever, and found the seat of trouble in the tubes and ovaries. In all his experience he had never seen pus in the pelvis independent of disease of the appendages. To make the statement definite, he had seen more than once double pyosalpinx and double ovarian abscess contained in a pus-pocket in the peritoneal cavity composed of adherent intestines and inflammatory tissue, four abscess cavities contained within a fifth. Again, he had seen a single pus tube with four distinct pockets in it. Pus can burrow through the cellular tissue and find vent as before stated. How shall pus in the pelvis be treated? The general principles of surgery for the treatment of pus in any other part of the body apply with equal force to the pelvis. Namely, where pus is present evacuate it, and, secondly, remove the cause of the suppurative process. It is equally as unsurgical and unscientific to allow pus to remain in the pelvis, as it would be to allow it to remain in the brain, in the mammary gland or under the fascias in any part of the body. It is equally as unsurgical to allow a suppurating tube or ovary to remain in the pelvis, as it would be to allow a sequestrum of dead bone, or to allow a necrotic placenta or membrane to remain in the uterus. These principles do not admit of evasion. All sorts and kinds of treatment have been tried without avail. Every man of experience knows the futility of counter-irritation, local depletion, or a general systemic treatment in the vast majority of cases.

There are only three methods of treatment common to physicians to-day—namely, electricity, vaginal drainage and abdominal section with the removal of the diseased parts, through irrigation of the peritoneal cavity and drainage.

The first of these methods need scarcely be mentioned in cases where pus is already present. Electricity has no place in the treatment of pus in the pelvis. Vaginal drainage is a crude, inefficient method, and is not so safe as some would have us believe. In abdominal section we have the quickest, easiest, most exact and therefore safest mode of treatment for pus in the pelvis. A small incision, rapid enucleation of the offending tubes and ovaries, the breaking up and evacuating of the separate pus-pockets, the separation of adhesions, the thorough washing out of the peritoneal cavity by copious irrigations of warm distilled water, the placing of a glass drainage tube in the most dependent portion of the peritoneal cavity, and the careful closure of the abdominal incision gives the patient the quickest relief, permanent cure, and very often snatches her from an impending death. Moreover, here we obtain the most ideal treatment, for at no other point of the body can we enucleate completely an abscess with its containing walls and pyogenic membrane. However, we should always bear in mind that the province of the surgeon is first, to save life; then to relieve suffering, rather than to perform ideal operations. Many patients dying with pus in the pelvis need but a feather's weight to depress the beam. In such cases the indications are to evacuate the pus, wash out the cavity and wait until a future time to remove the offending cause.

Paper discussed by Drs. Hardon, Engelmann, Stone, Robinson, Hadra and Potter.

Dr. W. L. Robinson read a paper on

GYNECOLOGY IN ITS RELATION TO OBSTETRICS, in which he spoke of the cervix uteri in its pathological condition, predisposing to hemorrhage prior to labor, laceration and septic absorption. He could find no explanation in medical literature of the cause of ulceration of the cervix, non-specific and non-malignant, causing hemorrhage in the cases which came under his observation within the last twelve months. He used the word "non-specific," because of the perfect health of patients prior to pregnancy and since delivery. The first case reported by him was seven months advanced in pregnancy and spending summer in the mountains for the benefit of one of her children. She found herself unwell after a week, and the flow was sufficient to soil the clothing and require napkins. A physician was called, who gave ergot and opium, which controlled the hemorrhage in twenty-four hours. Two weeks subsequently the conditions repeated themselves, and relief was obtained by the same remedies. She returned to the city and was again annoyed by a like hemorrhage, which came on about 7 P. M. daily, in spite of the efforts of the attending physician to control it, which continued until the sixth day, the patient having reached home. Dr. Robinson was now called, and made an examination with speculum. He found the os granular denuded of its mucous coat, and upon gently opening the os with uterine dilators, he discovered a small clot adherent to the ulcer, which caused bleeding when removed. He applied carbolic acid to ulcer, and dusted the os and vagina with boric acid. He continued treatment until the parts were in a healthy condition, and delivered the woman at full term without hemorrhage, tear or any unusual sequelæ. No placenta previa existed, and not one drop of blood escaped after the first application of carbolic acid until after delivery, and then everything was normal. The second case occurred a few weeks subsequent to the first, with history and result similar.

He has, for several years, made it a practice to examine his regular patients whenever a yellowish or dirty white vaginal discharge exists, especially if the vulva is in a state of irritation, and almost invariably finds the os granulated, whether lacerated or not, and he persistently treats such cases until restored to a healthy condition, explaining fully to the patient the importance of such treatment.

Dr. Bedford Brown, of Alexandria, Va., corroborated the statements of Dr. Robinson by the citation of a case. The patient had a bilateral laceration of the cervix. She became hostile to sexual intercourse with her husband, and had an intense dislike of his company and presence. This preyed upon her mind to such an extent that she became insane. He treated the case with applications of nitrate of silver, and the lacerations healed perfectly. After this all symptoms entirely disappeared. The patient regained her reason, her affection for her husband, and had lost the hostility to sexual intercourse which she had had. She has since borne three children. Dr. Brown has examined the condition of the cervix after each birth, and the repair is perfect.

The paper was further discussed by Dr. Richard Douglas, of Nashville.

FIRST DAY—EVENING SESSION.

The Association met in Broad Street Museum Hall at 8 P. M.

An Address of Welcome was delivered by Hon. A. J. Caldwell, the response to which was made by Dr. J. M. Mathews, of Louisville.

Dr. Hunter McGuire then delivered the Presidential Address, which was a scholarly, timely production, and was enthusiastically received.

SECOND DAY—MORNING SESSION.

Dr. Geo. J. Engelmann, of St. Louis, read a paper entitled

MENSTRUATION AND PREGNANCY AFTER REMOVAL OF BOTH OVARIES.

The following are the conclusions drawn from the history and microscopical examination of Dr. Engelmann's cases, which are corroborated by numerous cases of oophorectomy and double ovariotomy now observed, whose histories have been recorded for a sufficient length of time after the operation.

1. That the continuance of menstruation after removal of both ovaries is due to remnants of ovarian stroma left *in situ*.

2. That portions of the ovarian tissues, however small, which remain after the removal of the greater portion of the organ, whether or not the Fallopian tube be preserved, may retain their activity and continue the functions of the entire organ.

3. Even elongated pedicles may contain ovarian stroma in which the functional activity of the organ may be continued.

4. That remnants of ovarian stroma do not necessarily preserve their vitality and functional activity.

The deductions of practical value to the operator are even of greater importance, and they are these:

(a) For the successful performance of oophorectomy it is requisite that every particle of ovarian stroma shall be removed if the desired result is to be expected with certainty.

(b) If shrinkage of fibromas, the limitation of hemorrhage or cessation of annoying symptoms is to be accomplished with the greatest certainty, both ovaries must be completely removed, and not even a particle of ovarian tissue left *in situ*.

(c) In the performance of double oophorectomy in women not yet beyond the climacteric, and not suffering from utero-ovarian reflexes, such healthy ovarian tissue as may exist should be spared in order that functional activity may not be impaired.

Dr. W. D. Haggard, of Nashville, said it is very rare for a woman to menstruate regularly after removal of both ovaries and both tubes. He believed that the hemorrhagic discharges from the uterus after oophorectomy depended upon some other cause than that of menstruation. It may depend upon some trouble connected with the endometrium, as suggested by Dr. Engelmann, a polypoid growth or a congested condition of the blood vessels which supply the endometrium. In January last he removed both ovaries and both tubes in a woman, and it is barely possible that bits of ovarian stroma were left behind, as three months later the patient continued to have hemorrhagic discharges which greatly annoyed her.

Dr. A. W. Johnstone, of Danville, Kentucky, held and believed that the ovary has no more to do with menstruation than the clitoris has. To demonstrate this he had left ovarian tissue behind, yet menstruation had ceased. He gave at considerable length his reasons for such a belief.

Dr. Virgil O. Hardon, of Atlanta, Georgia, had operated on a patient about eighteen months since for a bleeding fibroid tumor, removing both ovaries, and in removing the second ovary he feared he had not removed all the ovarian tissue, as the precarious condition of the patient at the time would not permit a continuance or prolongation of the operation. The patient recovered from the operation and has menstruated with unvarying regularity from that time to the present.

Dr. Richard Douglas, of Nashville, said that Battey's operation of itself does not control menstruation, whereas in Professor Tait's operation, which consists in the removal of both ovaries and both tubes, the gynecologist embraced in his ligature the nerve which controls the menses.

Dr. A. V. L. Brokaw, of St. Louis, warmly took exception to some remarks made by Dr. Johnstone, who was inclined to give Tait the credit of first performing oophorectomy. He said he admired Tait's skill as an operator, but as a man he did not, for with characteristic English modesty he (Tait) adds his name to operations that do not rightfully belong to him; as, for instance the flap splitting operation.

Dr. Brokaw's remarks were warmly applauded by the Association.

Dr. W. H. Wathen, of Louisville, said gynecologists were more familiar with the laws that govern menstruation in many respects than they formerly were, but that we still require more scientific investigation, personal observation and experience to convince us that any one cause controls menstruation.

Dr. A. W. Johnstone said he arose for the purpose of defending an absent friend. The statement made by Dr. Brokaw that Tait was the originator of the flap-splitting operation is not true.

A full description of the method could be found in a recent issue of *Mund's Journal.* It is true that Tait had used the flap-splitting operation without knowing it had been described some twenty years ago by a Dublin surgeon, but in this issue he gives the originator due credit for the operation.

The paper was further discussed by Drs. G. Frank Lydston, and Johnstone, and discussion closed by Dr. Engelmann.

Dr. John D. S. Davis, of Birmingham, Alabama, then read a paper on

AN EXPERIMENTAL STUDY OF INTESTINAL ANASTOMOSIS, in which he reported thirty-two adhesive experiments on dogs and seventy-nine successful anastomotic operations by means of his approximation cat-gut mats and cat-gut plates for the purpose of illustrating the advantages of denuding the coaptation serous surfaces, and the integrity and absorbability of his cat-gut mats and plates.

He reported two applications of anastomosis to man. The first, ileo-colostomy for obstruction in the region of the ileo-cæcal valve, by means of cat-gut mats; the second, jejuno-jejunostomy for multiple gunshot injuries of the jejunum, with resection and lateral approximation by means of cat-gut plates.

His paper was replete with suggestive advantages of anastomosis over circular enterorrhaphy, based on experimental facts. His anastomotic devices consist of cat-gut mats and cat-gut plates —oval and horse-shoe. The mats are made of cat-gut in the following manner: A large continuous four-rib cat-gut frame is held in an oblong shape by four artery forceps, while the frame is being interwoven into an oval mat, of the desired size, by means of a small cat-gut thread, armed with a needle. The coaptation threads are fixed by passing a needle and thread between the two middle ribs and so returned as to loop two or three of the small gut sutures used in weaving the ribs together. The plates are made of any size by means of an ordinary pocket-knife, from a large one-eighth inch thick dry compressed plate of the uncut gut tissue—made for the author by William Snowden, Philadelphia. The coaptation threads are fixed by passing them through the plates by means of a needle, or better, by means of an awl and knotted to fix them. The horse-shoe plates are made from the oval plates by cutting out one end of each of the oval plates. They are used for closing in a hinge manner, extensive gunshot wounds of the convexity of the bowel.

His paper closed with the following propositions:

1. Approximation cat-gut mats may be made of any size, in less than an hour.

2. Approximation cat-gut plates may be made of any size, in from ten to fifteen minutes.

3. Approximation cat-gut horse-shoe plates are very valuable in intestinal repair from gunshot injuries of the convexity of the bowel.

4. Approximation cat-gut mats and plates absorb away in from forty-eight to sixty hours in gastro-enterostomy, and in from seventy to eighty hours in operation below the stomach.

5. Anastomosis, by means of approximation cat-gut mats or plates furnishes the best conditions for the healing of the visceral wound.

6. Anastomosis, can be performed by means of cat-gut mats or plates, without division of bowel, in five minutes; and with division or resection in fifteen minutes, including a continuous outside safety-silk suture around the circumference of the mats or plates.

7. Denuding the peritoneum of endothelium at the seat or coaptation hastens the exudation of plastic lymph, the formation of adhesions and the definite healing of the intestinal wound.

8. When coaptation serous surfaces have been denuded of their endothelial covering by mechanical scraping, plastic adhesions readily take place, and definite healing—by the formation of a network of new blood vessels in the product of tissue proliferation from the coaptation serous surfaces—is initiated in eighteen hours.

Dr. Davis' paper was followed by one entitled

### INTESTINAL ANASTOMOTIC OPERATIONS WITH SEGMENTED RUBBER RINGS, WITH SOME PRACTICAL SUGGESTIONS AS TO THEIR USE IN OTHER SURGICAL PROCEDURES, by Dr. A. V. L. Brokaw, of St. Louis, Mo.

For many months the author has been experimenting with segmented rubber rings in all the anastomotic operations, and such operations as gastrostomy, cholecystotomy duodeno-cholecystotomy, jejuno-cholecystotomy, and circular enterorrhaphy. The rings used by him are rapidly made, during an operation if necessary. All that is required is some rubber tubing or a soft ordinary rubber catheter and some cat-gut. He prefers tubing one-sixteenth to one-eighth of an inch in diameter. A section of this, of sufficient length to make a ring of the desired aperture, is cut into four or eight segments. Passing heavy strands of cat-gut through the lumen of these pieces, the ends are tied tightly enough to bring the ends of all segments together forming an oval ring. To the cat-gut strands are tied from four to six silk apposition of threads twelve to fourteen inches long, and the attachment of needles to these threads renders the ring ready for use. Another method is to pass a heavy double strand of cat-gut continuously through the segments several times; approximate the ends of the segments and push the ends of the cat-gut into the tubing. This ring will have a better surgical finish, and after the apposition threads are tied between the segments the ring will maintain its perfect form until the cat-gut is absorbed. The rings were passed as early as the fifth day in one of his experiments. In forming an amastomosis, after ordinary No. 6 darning needles are attached to the apposition thread, compress the ring and pass it through the opening made in the lumen of the bowel; then pass the threads through the intestinal wall from within outward. Ascertaining that the ring rests well in place, proceed to the second in the same manner; appose, and after scarification of the marginal serous surfaces, as suggested by Senn, tie the apposition threads. When possible, it is well to utilize omental grafts which add to the security. With two such rings circular enterorrhaphy may be performed, the rings corresponding in size to the lumen of the bowel, care being taken that they are not so large as to press too much upon the delicate mucosa or to overstretch the bowel, as a local gangrene might then follow. Introducing a ring at each end of the gut at the point of section, the threads are passed through the wall less than one-third of an inch from the divided margins. The distal end of the gut is invaginated and the proximal gut pushed into the distal, bringing the serous surfaces in contact. The threads are then tied and a few Lembert sutures added, the entire operation requiring less than ten minutes. In one-half of his experiments with this operation the result was excellent. Of the fourteen dogs operated on by this method, in seven the results were all that could be desired ; marked stenosis was found in several cases and in all a ridge at the seat of the operation.

In a recent paper the essayist mentioned a new procedure of closing large wounds of the intestine, especially gunshot, whereby ordinary suturing stenosis would result. This method applies to wounds of the surface of the intestine ; those of the mesenteric portion usually require resection. By this simple method wounds the size of a half dollar may be closed in less than five minutes. The wound being trimmed and enlarged with scissors, a ring two and a half inches in diameter, made of eight segments of tubing, with six apposition threads, two on each side, so arranged that when the apposition threads are tied the ring is held bent evenly on itself. Such a ring is introduced into the bowel, the end apposition threads passed, then the lateral, using a single cat-gut suture in drawing the wound margins together at the point of flexure in order to prevent eversion. A few Lembert sutures complete the operation. If two wounds are close together in the same loop, a lateral anastomosis might be formed if possible. With more than two wounds close together, excision and lateral anastomosis will require less time than circular enterorrhaphy or the sewing up of several wounds. Other conditions where the single ring may be used are perforating ulcers, fistulæ, etc. The great advantage of the segmented rubber rings over other devices is the simplicity of their construction and the rapidity with which any number may be made. The large aperture of segmented rings makes it possible to perform ileo-colostomy by the following method, which the author believes is original : The ileum being divided a short distance from the cæcum, the divided end of the distal bowel is invaginated into itself and secured by a continuous suture through the serous and muscular coats. Above the proximal end a clamp is placed and a ring adjusted to the lumen ; a slit is then made in the convex surface of the ascending colon and a ring introduced. The bleeding checked, the proximal end of the divided ileum is inserted into this slit, the threads tied and Lembert sutures added. This operaion may be quickly performed, and is indicated in cases as irreducible intussusception of the ileum into the cæcum and malignant diseases of the colon.

Appended is a series of operations with the results :

Gastrostomy.—Two experiments ; two recoveries.

Gastro-jejunostomy.—Three cases ; two recoveries ; one death from peritonitis. (Dog tore one suture eight days after operation.)

Jejuno-ileostomy.—One case ; result perfect.

Ileo-ileostomy.—Two cases ; one death, due to perforative peritonitis.

Ileo-colostomy.—Two cases ; two perfect results.

Colo-colostomy.—Three cases ; one death.

Ileo-rectostomy.—Two cases ; perfect results.

Circular enterorrhaphy.—Fourteen cases ; seven deaths.

Duodeno-cholecystotomy.—Three cases ; two deaths from peritonitis. (This operation is difficult to perform on a dog for anatomical reasons.)

Partial duodenectomy.—Two cases ; one perfect result.

Partial jejunectomy.—Two cases ; two perfect results.

Partial ileectomy.—Four cases ; one death.

Partial colectomy.—Two cases ; two perfect results.

SUMMARY.—Intestinal anastomotic operations.—Fourteen cases ; three deaths.

Circular enterorrhaphy.—Fourteen cases ; two deaths.

The single ring formed of eight segments of tubing was used in closing wounds varying in size from a quarter to half a dollar in nine cases with one death. The clamp devised by him and used in the operations is made of No. 12 copper wire, covered with unperforated rubber tubing of small size.

SECOND DAY—AFTERNOON SESSION.

The papers of Drs. Davis and Brokaw were discussed conjointly by Drs. W. O. Roberts, Virgil O. Hardon, B. E. Hadra, A. M. Owen, of Evansville, Indiana, Hunter McGuire, W. W. Potter, R. S. Cunningham, G. Frank Lydston, Richard Douglas, Geo. J. Engelmann, W. E. B. Davis, and discussion closed by the essayists.

Dr. B. E. Hadra, of Galveston, Texas, read a paper entitled

THE OPEN ABDOMINAL TREATMENT, in which he said that abdominal surgery, notwithstanding its immense progress, has not as yet given even a moderate degree of satisfaction in acute diffuse septic peritonitis. Chronic infectious processes offer much better prospects for surgical interference, such as tuberculosis, actinomycosis, and the recently described microbic peritonitis of as yet unknown origin.

The points making the diffuse septic peritonitis are :

1. The extensive area of peritoneal surface with its enormous power of resorption of the poisonous fluid.

2. The active secretion into the sac and thereby furnishing cultivating fluids for the germs.

3. The ready absorption by the lymphatics of the diaphragm.

4. General distribution of the poison by intestinal peristalsis.

5. The infection of the intestinal walls from without, and the additional infection of the peritoneal cavity by transudation and immigration of germs from the inside of the bowels.

6. Distension of the bowels, increasing the pressure and resorption.

7. The impeding effect of this latter condition upon respiration, defecation and secretion of urine, leading to systemic poisoning by retained products of oxidation.

8. In perforative cases the contamination by fæcal matter; in stab and gunshot wounds by other impurities, bile, urine, etc., and above all, contaminated blood.

The indications for treatment, besides supporting the patient's strength, relieving suffering, giving proper action to the bowels, kidneys —in short, besides the general medical treatment, are

1. To remove the obnoxious material—germs, fæcal matter, urine, etc.

2. To prevent its new formation or a repetition of its entrance. The sac should be kept dry to deprive the germ of its soil. The breaks have to be mended so that the channels of contamination may not lead to the outside.

3. To prevent the bowels from distributing the poison throughout the whole cavity.

4. To counteract pressure and suction in order to prevent resorption of the poison.

5. To prevent infection of the peritoneum and the bowel.

6. To relieve pressure in order to avoid disintegration and paralysis of the different structures.

7. To free respiration, defecation and urination with tympanites as developed.

Dr. L. S. McMurtry, of Danville, Kentucky, read a paper on

TWENTY CONSECUTIVE CASES OF ABDOMINAL SECTION.

The series of cases comprised the first twenty abdominal sections performed by him, and illustrated a variety of pathological conditions and diverse complications. All the cases were in private practice, and with two exceptions all the operations were done at the homes of the patients. Two cases were treated as private patients in a well appointed hospital. In many cases operative treatment was only accepted after all ordinary and so-called conservative measures had been exhausted; and in several cases the operation was only accepted when the patient's condition was regarded hopeless by both physician and family. In no case, however desperate and complicated, was an operation refused. One case was diagnosed by four different surgeons (before he saw the patient) as ovarian cyst, which illustrates the difficulty attending the diagnosis of intra-abdominal disease. The history and physical signs were characteristic of that condition. On opening the abdomen it was found to be encysted dropsy, the result of tubercular peritonitis. Evacuation and irrigation were followed by quick recovery, and though the peritoneum was studded everywhere with miliary tubercles, and the patient is now sixty-eight years of age, she remains to-day in perfect health and activity.

Another case of soft uterine myoma was also believed to be an ovarian cyst. The tumor was very soft; it was besides heavily œdematous and fluctuated distinctly. The history and general condition of the patient tallied with the deceptive diagnosis. It was only when the abdomen was opened that he had realized he was to do a supra-vaginal hysterectomy, instead of an ovariotomy. Seven of the cases recorded were ovariotomies. The cysts were in every instance large, and had, with two exceptions, been tapped several times. One was a suppurating cyst.

Dr. McMurtry said thorough work, irrigation and drainage all conjoined gave the only basis of success in the cases reported. In a number of his cases he had operated in the midst of active peritonitis, with vomiting and tympanites. In this condition of affairs he had witnessed the most gratifying results from persistent and oft-repeated exhibition of calomel, dropping two or three grains on the tongue every hour until the bowels were freely moved. Increasing experience has impressed him more and more with the difficulties of abdominal work, and makes him less confident of meeting often with simple cases.

He closed his paper with a plea for earlier interference in abdominal diseases. When operations are done in good time, before emaciation and exhaustion come, and before repeated attacks of peritonitis have complicated the camparitively easy task for the surgeon, then will the surgeon's results excel even the brilliant records of the present time.

Dr. Richard Douglas, of Nashville, read a paper entitled

### COMPLICATIONS OCCURRING IN THE CLINICAL HISTORY OF OVARIAN TUMORS.

The papers of Drs. Hadra, McMurtry and Douglas were discussed conjointly by Drs. Potter, Roberts, Stone, Wathen, Engelmann, Hardon, Haggard, Lydston, Cunningham, Brownrigg, W. E. B. Davis, and closed by the essayists.

SECOND DAY—EVENING SESSION.

Dr. G. Frank Lydston, of Chicago, Illinois, read a paper on

TROPHO-NEUROSI AS A FACTOR IN THE PHENOMENA OF SYPHILIS, in which he called attention to the relation of disturbances of the trophic function of the sympathetic nervous system, which he claimed were the essence of all the phenomena of syphilis. He said the relation of certain syphilitic phenomena to organic or functional disturbances of the nervous system, and particularly the sympathetic system, are manifested here and there along the whole line of morbid phenomena developed in the course of disease. Syphilitic fever is undoubtedly dependent upon the action of a special poison upon the sympathetic nervous system. From what we know of the trophic functions of the sympathetic, we are justified in inferring that the majority of fevers are dependent upon the action of a specific poison upon the sympathetic ganglia. The syphilitic poison may produce disturbance of the sympathetic with perversion of tissue metabolism and excessive production of heat. The inconstancy of the syphilitic fever is explicable upon the ground of idiosyncrasy. The syphilitic roseola has been demonstrated to be an exception to the rule that syphilitic lesions are due to a collection of proliferating cells. It is due to vaso-motor disturbance, with resulting dilatation of capillaries. This nervous disturbance is dependent upon the impression of the syphilitic poisons upon the sympathetic ganglia. The accumulation of cells in the more pronounced lesions of syphilis is simply an exaggeration of the normal process of tissue building. As is well known, such tissue building is presided over by the filaments of the sympathetic nerves.

The symmetry of the peripheral phenomena of syphilis is suggestive of some causal condition affecting the central nervous system. As an illustration of the manner in which a nerve lesion could produce disturbed nutrition the author mentioned herpes zoster. Some of the lesions of syphilis which are difficult of explanation upon mechanical grounds—*i. e.*, upon the theory of localized cell accumulation, are readily explicable by central or local nervous disturbance. For example, the alopecia of syphilis is similar to that which occurs in other diseases as a consequence of local and general malnutrition incidental to disturbed nervous supply—as, for instance, alopecia aræta, the alopecia produced by fevers, and the alopecia produced by neuralgic affections of the head. That the nutrition of the hair is profoundly affected by nervous disturbances is shown by the result of fright in producing blanching of the hair.

One of the principal arguments in favor of the theory tha tropho-neurosis is the foundation of syphlitic processes is the peculiar action of the disease when it attacks certain parts, syphilis seemingly possessing the power of dissecting out definite portions of osseous tissue (apparently by cutting off their nutritive supply) in a manner as cleanly as it can be done by the knife. Thus the essayist has in his possession specimens of the inferior maxillary bone, portions of the alveolar process of the maxilla, the palatal and nasal processes of the superior maxilla, the malar and ossæ nasi which became necrosed and were removed from cases of late syphilis. These fragments present as natural a conformation as in their healthy condition. The ordinary explanation of destruction by pressure of syphilitic exudate will not suffice in these cases. If they be observed carefully it will be found that the first symptoms experienced by the patient are those identical to the presence of a foreign body, i. e., dead bone in the tissues. If pressure were the cause of the necrosis the death of the bone would be preceded by more or less painful swelling and inflammation.

He claimed that all of the pathological processes incidental to syphilis are due to disturbances of nutrition produced by the impression of the syphilitic poison upon the sympathetic nervous system, and that it is immaterial to the cogency of this theory whether the poison of syphilis be a microbe, bacillus, degraded cell, or chemical poison. If any attempt has been made to show that tropho-neurosis is the basis of all syphilitic phenomena, the author is not aware of it.

THIRD DAY—MORNING SESSION.

Dr. John Brownrigg, of Columbus, Miss., read a paper on

GUNSHOT FRACTURES OF THE FEMUR, in which he discussed the class of cases requiring amputation, and those in which a more conservative course should be pursued. He exhibited several appliances devised by himself.

Dr. Hunter McGuire, of Richmond, Va., read a short paper on the

TREATMENT OF CYSTICS IN WOMEN, which was followed by a paper on the

TREATMENT OF CONTRACTED BLADDER BY HOT WATER DILATATION, by Dr. I. S. Stone, of Lincoln, Va.

During the past few years certain protracted cases of cystitis, occurring chiefly in women, have been observed by Dr. Stone, which have resisted all known forms of medical treatment, and necessitated some surgical or mechanical measure of relief.

He describes the manner of dilatation as follows: "The patient is given morphia sulphas gr. $\frac{1}{4}$, atropia sul. gr. $\frac{1}{200}$ hypodermically. She is placed on her back on a table for convenience, although it would answer to arrange the bed with the patient thereon to suit the operator. A soft catheter is at once inserted into the bladder, and after the urine has escaped, hot water, temperature 110, is thrown into the bladder, until the patient will no longer bear it. This is allowed to escape, and is measured, giving the full size of the bladder in its present condition. As the morphia gradually becomes absorbed, the patient will bear further distension, each time perhaps one drachm may be added to the capacity of the bladder. I prefer using a rubber ball syringe, holding two to four ounces. The pressure of the hand is safer than that of the tube or funnel or any instrumental gauge, as the patient generally is unable to resist the tendency to strain, owing to tenesmus produced by the expansion, As each seance should continue thirty to sixty minutes the bladder may be filled and emptied many times, and at first the operator must be well satisfied if the gain is only one or two drachms, in a bladder whose capacity is perhaps only two ounces. As the patient becomes fully under the influence of the morphia, the water may be increased in temperature to 120 or 120 F. The very best effects follow its use when at this temperature."

These papers were discussed by Drs. Lydston, Davis, Engelmann, Roberts, Brokaw and Hadra.

Dr. Bedford Brown, of Alexandria, Va., read an interesting paper entitled

REMARKS ON CERTAIN OBSCURE AND MINOR FORMS OF PELVIC CELLULITIS SIMULATING MALARIAL FEVER.

By referring to his note-books he found some twelve cases of pelvic cellulitis, some of a grave and others of minor form, but all so very obscure in their manifestations of the presence of local lesion as to be well calculated to mislead and to cause a mistaken or false diagnosis to be made of malarial fever. There was nothing in these cases to call the attention to the pelvic organs. He has in several instances seen these cases run their course from beginning to end without manifesting the first symptoms of local disease in the pelvis, so that the resemblance to malarial fever was very nearly complete.

Dr. Jos. Taber Johnson, of Washington, D. C., presented a paper entitled

OBSERVATIONS BASED UPON AN EXPERIENCE OF SEVENTY-TWO MISCELLANEOUS ABDOMINAL SECTIONS.

Of this number twenty-nine were for the removal of ovarian tumors varying in size from one to sixty-four pounds, twenty-six recoveries and three deaths; twenty-nine cases of removal of the uterine appendages, with twenty-seven recoveries and two deaths; seven supra-vaginal hysterectomies for large uterine fibroids, with three recoveries and four deaths; one Cæsarean section, death on the tenth day; one cyst of the kidney, weighing seventy-four pounds, died of exhaustion; one fatal case of extra-uterine pregnancy, operated on six weeks after rupture, general peritonitis with pulse 130, temperature 103, for the week previous; one fatal case of general abdominal cancer, three exploratory incisions, all recovered. Total, seventy-two laparotomies, with fifty-nine recoveries and thirteen deaths.

Of the fifty-eight ovarian operations the first three deaths were the second and third and fifth of his series. In the last fifty-two ovarian operations there have been only two deaths. One of these was from tetanus occurring on the fifteenth day after operation, when everything indicated a perfect recovery. The other was an insane patient who had been four years in an insane asylum on account of nymphomania. She could not be entirely controlled, and her efforts to get out of bed set up inflammation about the abdominal sutures, causing an abscess, which burst internally and caused death.

Dr. Johnson emphasized the statement that experience in operating was nowhere so valuable as in the abdominal cavity; that the "unexpected" was so often found, that many cases would be lost if the operator was not prepared for and equal to the emergencies as they "unexpectedly" arose.

The following papers were read by title:

1. Puerperal Eclampsia, by Dr. John Herbert Claiborne, Petersburg, Va.

2. Laparotomy in Intestinal Obstruction, by Dr. Cornelius Kollock, Cheraw, South Carolina.

3. The Causes of Frequent Failure of Relief of Reflex Symptoms after Trachleorrhaphy, by Dr. W. F. Hyer, Meridian, Miss.

The following officers were elected:

President—Dr. George J. Engelmann, of St. Louis, Mo.

First Vice-President—Dr. B. E. Hadra, Galveston, Texas.

Second Vice-President—Dr. Duncan Eve, Nashville, Tenn.

Secretary—Dr. W. E. B. Davis, of Birmingham, Ala.

Treasurer—Dr. Hardin P. Cochrane, of Birmingham, Ala.

On motion, the Association adjourned to meet in Atlanta, Ga., second Tuesday in November, 1890.

